# The Ambivalent Nature of *Bacteroides fragilis* and the Interaction with *Clostridioides difficile*: Benefits and Disadvantages for the Human Host

**DOI:** 10.3390/toxins17100513

**Published:** 2025-10-19

**Authors:** Patrizia Spigaglia

**Affiliations:** Department of Infectious Diseases, Istituto Superiore di Sanità, 00161 Rome, Italy; patrizia.spigaglia@iss.it

**Keywords:** *B. fragilis*, toxin, BFT, ETBF, NTBF, *C. difficile*, CDI, CRC, infection

## Abstract

*Bacteroides fragilis* is a usually beneficial colonizer of the human gut that can also act as an opportunistic pathogen, causing infection and contributing to the development and progression of important diseases. The production and secretion of the *B. fragilis* toxin (BFT), the main virulence factor of this bacterium, distinguishes enterotoxigenic (ETBF) from non-toxigenic (NTBF) strains. Although NTBF does not produce the BFT, certain strains can exhibit unexpected pathogenic characteristics. The complex interactions between *B. fragilis* and the other intestinal bacteria, such as *Clostridioides difficile*, the leading cause of antibiotic-associated diarrhea in healthcare settings, highlights its ambivalent role of benefactor and pathogen. In fact, although *B. fragilis* plays a part in preventing colonization and infection due to *C. difficile* (CDI), both these anaerobic bacteria can contribute to the development and progression of colorectal cancer (CRC), one of the most prevalent malignant tumors of the digestive tract. This review provides an overview of the dual nature of *B. fragilis*, focusing on the peculiarities of ETBF and NTBF, delving into *B. fragilis* interaction with *C. difficile* and impacts on the host.

## 1. Introduction

*Bacteroides fragilis* is a Gram-negative, rod-shaped anaerobic bacillus belonging to the family *Bacteroidaceae*, which accounts for about 1–14% of the *Bacteroides* species in human feces [1,2]. *B. fragilis* can establish commensal relationships with the human host, playing a key role in sustaining the microbial food web of the gut and providing the host with numerous health benefits, but it can also act as an opportunistic pathogen, causing intestinal and extraintestinal infections and contributing to the development and progression of some human diseases [1,3,4,5,6]. The production of a zinc-dependent metalloprotease, known as the *B. fragilis* toxin (BFT), distinguishes two broad categories of *B. fragilis* strains: enterotoxigenic *B. fragilis* (ETBF), which secrete the BFT, and non-toxigenic *B. fragilis* (NTBF), which do not produce the BFT [7].

Commensal *B. fragilis* provides benefits to the host since it can promote immune system maturation, suppress abnormal inflammation, and reduce various diseases, such as colitis, central nervous system disorders, bacterial infections, and cancer [8,9,10,11,12]. Nevertheless, ETBF strains are often correlated with anaerobic bacteremia, intra-abdominal abscesses, appendicitis, asthma, inflammatory diarrhea, inflammatory bowel disease, and lung abscess infections [13,14,15,16], while NTBF strains can act as opportunistic pathogens in other areas of the body different from the gut, contributing to different human diseases [7,17,18,19,20,21].

The ambivalent nature of *B. fragilis* towards the host is also shown in its interactions with other pathogens, including *Clostridioides difficile*. *C. difficile* (CD) is a Gram-positive, spore-forming, opportunistic anaerobic bacillus known to be the leading cause of antibiotic-associated diarrhea in hospital settings but also in the community [22]. *C. difficile* infection (CDI) ranges from self-limited diarrhea to pseudomembranous colitis and toxic megacolon, often characterized by high morbidity and mortality rates [22,23]. Interestingly, there is evidence that *B. fragilis* has a role in preventing CD colonization and infection [24], but also that both *B. fragilis* and CD contribute in different ways to the development and progression of colorectal cancer (CRC), one of the most prevalent malignant tumors in the digestive tract [25,26].

The aims of this review are to provide an overview of the ambivalent nature of *B. fragilis*, commensal and pathogen, focusing on the peculiarities of ETBF and NTBF strains, and delving into *B. fragilis* interaction with CD and the consequent benefits and disadvantages for the human host.

## 2. The *B. fragilis* Toxin (BFT)

The *B. fragilis* biologically active factor has been identified as a heat-labile, ~20 kDa protein, currently known as the *B. fragilis* toxin (BFT) [27,28,29]. The BFT is a member of the matrix metalloprotease subfamily of the metzincin superfamily of zinc-dependent metalloprotease enzymes, with a spectrum of biological activities [29,30]. The BFT has an important role in causing intestinal inflammation and injury by damaging the tight junctions of epithelial gut cells and increasing intestinal permeability [31,32]. The BFT also induces intestinal epithelial cells to express and secrete the pro-inflammatory chemokine interleukin-8 (IL-8), a chemokine that attracts polymorphonuclear cells and concurs in the colonic inflammatory induction [33,34].

The *bft* gene is carried by a ~6 kbp transposable element, the *B. fragilis* pathogenicity island (BfPAI) [35,36]. The BfPAI is flanked by genetic regions encoding mobilization proteins involved in the modulation of the *bft* expression [37,38]. Three different BFT variants have been identified: BFT1, BFT2, and BFT3 [39,40]. Data available show heterogeneity in the prevalence of the BFT variants in clinical samples, indicating the BFT1 and the BFT2 as the most frequently detected [41,42,43]. Studies on population-wide carriage demonstrate that the BFT1 is geographically the most widely distributed, while the BFT3 seems to be restricted to the southeast part of Asia [43,44,45,46]. Experiments in vitro and in germ-free mice demonstrate different degrees of potency among BFT variants, with the BFT2 exhibiting the greatest potential to damage intestinal tissue [46,47].

Wu et al. have demonstrated that the BFT binds to intestinal epithelial cell lines in vitro only at 37 C°, and that the BFT binding is resistant to acid washing, suggesting an irreversible interaction [48]. Furthermore, in the same study, protease-activated receptors (PARs) have been identified in vitro as BFT receptors on the intestinal epithelial cells but with no effects on the biological activity of the BFT, which probably needs other intestinal epithelial cell receptors.

The BFT activity occurs by modification of cell surface molecules, principally cleaving the E-cadherin protein of the zonula adherens, with a degradation of both the extracellular and intracellular domains of this protein [49]. The extracellular region of the E-cadherin has a role in cell-to-cell interaction, while the intracellular region, through its association with α-catenin and β-catenin, connects to the actin cytoskeleton of epithelial cells [50]. The complete degradation of the E-cadherin leads to loss of cell–cell contacts and cell rounding, altering the function of ion transporters and triggering induction of mitogen-activated protein kinases (MAPKs) and the nuclear factor kappa-light-chain-enhancer of activated B cells (NF-kB) pathway, thus increasing secretion of IL-8 [33,34,51,52,53]. Furthermore, when the E-cadherin is degraded, more soluble β-catenin becomes available for the Wnt/β-catenin signaling, inducing a cell proliferative response through the c-myc protein [50]. Some studies have reported that histological analysis of the guts of specific-pathogen-free (SPF) mice with acute colitis due to the BFT shows rupture of cell–cell adhesions and epithelial exfoliation, with the presence of immune cell infiltrates, two days after infection [54], while colonic histology of chronic colitis shows progressive hyperplasia of the colonic crypts, consequent with BFT-dependent induction of a program of cell hyperproliferation, between 7 days and up to 16 months after infection [54,55].

Among *B. fragilis* strains, ETBF strains contains a BfPAI and produces the BFT, while NTBF strains do not show a BfPAI or present only the BfPAI flanking regions and, therefore, do not produce the BFT [7,36,56].

## 3. *B. fragilis*: Commensal and Pathogen

Both NTBF and ETBF strains can act as beneficial commensals for humans while also possessing pathogenic potential, causing infection not only in the gastrointestinal tract but also in other sites of the body when they breach the gut barrier due to damage to its integrity (i.e., rupture of an inflamed appendix, intestinal surgery, physical injury, etc.). Furthermore, there is evidence that *B. fragilis* also plays a role in the development and progression of human diseases, such as neurodegeneration and cancer.

### 3.1. Commensal Role

*B. fragilis* colonized the human gut in the neonatal period, from the mother and other individuals closely interacting with the newborn [57,58]. Several factors, such as gestational age, birth and feeding patterns, birth environment, and ethnic/geographic background, have a role in the colonization of the gut of newborns by *B. fragilis* [59]; for example, higher levels of *B. fragilis* have been reported in feces samples of vaginally delivered infants [60].

Under normal circumstances, *B. fragilis* colonization is benign, with potential benefits for the human host [61,62] (Table 1).

As a component of the gut microbiota, *B. fragilis* breakdowns polysaccharides of plants that are not degraded by the human digestive tract, producing short-chain fatty acids that represent a source of energy for the intestinal epithelium [5].

*B. fragilis* colonization is usually associated with benefits for the host that include immune regulation and anti-inflammatory effects [62]. The polysaccharide A (PSA) of this bacterium exhibits significant pro- and anti-inflammatory properties that are T cell dependent [63]. The *B. fragilis* PSA can suppress inflammatory bowel disease and experimental autoimmune encephalomyelitis (EAE) through activating helper T cells that express the surface protein CD4 (CD4+ T cells) through a mechanism dependent on the major histocompatibility complex class II (MHCII) [63]. Interestingly, a *B. fragilis* enrichment in centenarians seems to enhance longevity due to an up-regulation of the anti-inflammatory factor IL-10, produced by regulatory CD4+ and CD8+ T cells, that regulates the critical balance between well-being and illness [64].

A diet containing larger quantities of meat and carbohydrates has been associated with an increased prevalence of *B. fragilis* in the gut, while a low-fat vegan diet has been associated with a decrease in the *B. fragilis* abundance [65,66]. Furthermore, some physical conditions of the host and the use of drugs can also affect the prevalence of *B. fragilis*. Recent studies have reported that in the gut of both individuals suffering from Alzheimer’s disease [67] and patients with diagnosed type 2 diabetes (T2D), after treatment with metformin [68], *B. fragilis* has been found less abundant than in healthy individuals. Metformin primarily acts by activating the protein kinase (AMPK) in the liver, decreasing gluconeogenesis and increasing the peripheral glucose uptake, but, recently, the hyperglycemia-lowering action of metformin has also been associated with a modulation of the components of gut microbiota [69]. Sun et al. have observed that metformin partially acts through a *B. fragilis*-bile acid glycoursodeoxycholic acid (GUDCA)-intestinal farnesoid X receptor (FXR) axis to improve metabolic dysfunction, such as hyperglycemia [68]. In their study, it has been observed that in the guts of patients with T2D, naively treated with metformin for three days, *B. fragilis* decreased, while GUDCA increased, with an inhibition of FXR signaling and an improvement of various metabolic endpoint dysfunctions, including hyperglycemia. Evidence suggests that FXR, regulating genes involved in the secretion of antimicrobial compounds and the integrity of the intestinal barrier, could play a role in suppressing bacterial overgrowth [69]. Furthermore, a recent study has reported that metformin inhibits the growth of *B. fragilis* through modification of folate and methionine metabolism, although with a mechanism that is still unclear [68]. More quality studies and standardized protocols are necessary to fully elucidate the underlying mechanisms of metformin interaction with gut bacteria and the clinical significance of its influence on certain groups of these bacteria.

#### 3.1.1. Non-Toxigenic *B. fragilis* (NTBF)

Commensal NTBF strains have potential health benefits for the human host. Using an experimental EAE mouse model, Ochoa-Repáraz et al. have shown that the purified PSA of the NTBF strain NCTC 9343 serves as both a preventive measure and remedy for changes in gut microbiota, as well as to prevent disease progression, after treatment with oral antibiotics [70] (Table 1). Wang et al. found that the PSA of B. fragilis might hinder the development of EAE by toll-like receptor 2 (TLR2)-triggered CD39 signaling and that the expression of the ectonucleotidase CD39 on CD4+ T cells is indeed associated with an equilibrium between Th17 cells, secreting IL-17, and CD4+ T cells, secreting IL-10 [8]. Other studies have reported that the PSA of NTBF can alleviate airway inflammation and experimental asthma by inducing CD4+ T cell proliferation and IL-10 synthesis [10,71]. Specifically, effector T cells, exposed to the PSA of NTBF, induce the production of IL-10 by the transcription factor forkhead box P3 (Foxp3) regulatory T cells, leading to mitigation of pulmonary inflammation [10]. Using animal models, Zhang et al. have also observed that oral administration of the NTBF strain ATCC 25285 (also denominated NTCC 9343) reduces inflammation by increasing regulatory T cells, inhibiting the development of atrial fibrillation in aging rats [72]. Interestingly, when the NTBF strain ATCC 25285 has been introduced into MRL/lpr mice, a rebalancing of Th17/regulatory T-cell levels has been observed, in accordance with the reported impact of B. fragilis on various autoimmune conditions [73].

Finally, NTBF has been reported to have a role in protecting and alleviating symptoms due to human pathogens, such as *Helicobacter hepaticus* [74], *Salmonella heidelberg* [75], *Vibrio parahaemolyticus* [76], and *Cronobacter sakazakii* [77].

#### 3.1.2. Enterotoxigenic *B. fragilis* (ETBF)

ETBF strains have been isolated in stools from both healthy individuals and patients with diarrhea, with higher prevalence values in diarrheal patients. In fact, studies indicate that ETBF is found in approximately 20–30% of diarrheal patients, while in healthy individuals the prevalence can range from 10–20%, suggesting a strong association between ETBF and diarrheal illness [78,79,80].

In young children, especially those younger than 1 year, ETBF prevalence is low, and it is not typically associated with diarrhea, while in older children (>1 year) ETBF colonization rates are higher and frequently associated with diarrhea, suggesting that the age of children is important for diarrheal association to occur [78,81,82,83,84].

It is still unclear what could be the benefit of ETBF commensal for the human host, as well as if these strains produce the BFT in asymptomatic carriers. It is possible that during gut homeostasis, either the BFT production could be abolished or become non-damaging, while perturbations of the gut homeostasis could lead to a susceptibility of the host to ETBF, resulting in inflammation and dysbiosis [48] (Table 1). The production of BFT might facilitate the survival of ETBF strains in the gut, conferring a selective advantage, as observed for *Vibrio cholerae* [85]. In fact, the cholera toxin CTX modulates the host cell metabolism, creating an iron-depleted intestinal niche that allows this bacterium to grow by acquisition of host-derived nutrients, including heme and long-chain fatty acids (LCFAs) [85].

Casterline et al. have observed that an ETBF strain can colonize a colonic niche previously occupied by an NTBF strain in a toxin-dependent manner [56], although niche acquisition or competition also relies in part on the *B. fragilis* type VI secretion system (T6SS) and other genetic determinants [86,87,88].

### 3.2. Pathogenic Role

Microbial and human host factors that, temporally and spatially, control the plasticity of the *B. fragilis* niche and that are responsible for progression from *B. fragilis* asymptomatic carriage toward infection remain in large part undefined [49].

A recent genomic analysis of numerous *B. fragilis* strains has shown new insights into the genomic underpinnings that facilitate *B. fragilis* transition from commensal to pathogen, showing that this bacterium has an expansive pangenome characterized by extensive genetic diversity, supporting both its adaptability and pathogenic potential [4] (Table 1). Interestingly, several *B. fragilis* virulence factors also have a role in both immune regulation and colonization of the gut, suggesting that these factors could be essential for survival/adaptation within specific environmental contexts rather than exclusively mediating pathogenicity. The presence of an open pangenome and the possibility to acquire the BFT via BfPAI horizontal transfer support that *B. fragilis* commensal strains continuously integrate new genetic elements, acting as reservoirs for determinants of virulence and contributing to the emergence of pathogenic variants [4,89].

*B. fragilis* capsule shields this bacterium from both complement-mediated killing and phagocytic uptake and killing by the host’s immune system [90,91,92]. Interestingly, *B. fragilis* can produce at least eight distinct capsular polysaccharides, regulating expression of genes in an “on-off” manner by the reversible inversion of the DNA segment containing the promoters, with a consequent modulation of its surface antigenicity, which facilitates evasion from the host immune response [93,94]. In addition, Vieira et al. have reported that *B. fragilis* can interfere with the peritoneal macrophages, the first host immunologic defense response to rupture of the intestine or other compromise of the peritoneal cavity [95].

#### 3.2.1. Non-Toxigenic *B. fragilis* (NTBF)

Certain NTBF strains have an increased propensity to elude the human host’s defenses and migrate from intestinal tissue, causing abscesses by bacterial translocation [7] (Table 1). It has been observed that the purified capsular polysaccharides of the NTBF strain NCTC 9343 can induce intra-abdominal abscess development in animal models [94,96]. Compositional and immunochemical analysis of the capsular polysaccharides of another NTBF strain, the *B. fragilis* 638 R, has identified a zwitterionic charge motif that correlates with the ability of capsular polysaccharides to induce experimental intra-abdominal abscesses [97].

NTBF strains can also aggravate the progression of metabolic diseases through bile acid metabolism [7,98]. In fact, NTBF bile salt hydrolase (BSH), altering bile acid composition and signaling pathways, potentially contributes to the development and progression of metabolic disorders, such as obesity and diabetes [99,100,101]. Pumbwe et al. have observed that both conjugated and unconjugated bile salts stimulate an overproduction of fimbriae and outer membrane vesicles in the strain NCTC 9343, along with a stimulation in the expression of N-type efflux pumps and the outer membrane protein OmpA [102]. This stimulation leads to a consequent increase in the antimicrobial resistance of this strain, as well as in its capability of aggregation, formation of biofilm, and adhesion to cells of the intestinal epithelium, thereby influencing interactions between NCTC 9343 and the host.

Using animal models, it has been demonstrated that the NTBF strain ATCC 25285 can induce intrahepatic cholestasis (ICP) through the activity of its bile salt hydrolase (BSH), which inhibits the farnesoid X receptor (FXR) leading to an excessive synthesis of bile acids, interrupting hepatic bile excretion, and ultimately promoting the initiation of ICP in pregnancy patients [103]. NTBF strains have also been associated with severe illness in individuals with diabetes. Sofi et al. have reported that the introduction of NTBF strain ATCC 25285 in nonobese diabetic (NOD) mice led to enhanced gut permeability, accelerated disease progression, and rapid onset of hyperglycemia [104].

In a study on the potential role of *B. fragilis* in inducing chronic low-grade inflammation and contributing to atherosclerotic cardiovascular disease, Shi et al. have found that the introduction of the NTBF strain ATCC 25285 in apolipoprotein E knockout (Aope^−/−^) mice results in an imbalance of the gut microbiota [105]. Dysbiosis has been characterized by a reduction in the prevalence *Lactobacillaceae* and an increase in the prevalence of *Desulfovibrionaceae*, which aggravates glucose and lipid metabolic disorders and the inflammatory response, and ultimately accelerated plaque formation and atherosclerosis progression in the aorta.

#### 3.2.2. Enterotoxigenic *B. fragilis* (ETBF)

The first study reporting ETBF isolation from humans with diarrheal illnesses dates to 1987 [106], and an association between ETBF and human diarrheal disease was demonstrated in pediatric patients in 1992 [78]. ETBF gastrointestinal infections are typically characterized as self-limited watery diarrhea, although up to 22% of patients may experience persistent diarrhea lasting > 14 days, which is clinically indistinguishable from diarrhea due to other pathogens [80,106,107]. ETBF strains induce inflammatory diarrhea, and this is supported by the detection of immunoglobulin A (IgA) and IgG antibodies against the BFT in serum and IgA in feces [108]. Furthermore, experiments in vitro have demonstrated that the BFT can rapidly and irreversibly intoxicate human intestinal epithelial cells (HT29/C1) [109].

ETBF strains have also been investigated as a cause of nosocomial and intra-abdominal abscesses. Cohen et al. have reported a statistically significant difference in the ETBF prevalence between hospitalized patients with diarrhea and healthy controls (26.8% vs. 12.4%, respectively), suggesting an association [110]. Sears et al. have also reported ETBF to cause a clinical syndrome with nonfebrile inflammatory diarrhea and marked abdominal pain in both hospitalized children and adults [111].

Gastrointestinal infection by *B. fragilis* usually initiates with a gross contamination of the peritoneal cavity, which progresses to a limited polymicrobial infection with fibrin abscess formation [1,7]. The abscess, a fibrous membrane that localizes invading bacteria and surrounds a mass of cellular debris, dead polymorphonuclear leukocytes, and a mixed population of bacteria, is a pathological host response to the invading *B. fragilis* [20]. Abscess formation has been linked to the *B. fragilis* capsule in an animal model, and injection of capsules alone has been demonstrated to be sufficient to induce abscess formation [20]. Abscesses left untreated can expand, causing intestinal obstruction, erosion of resident blood vessels, and ultimately fistula formation or rupture, causing bacteremia [1].

In healthy individuals, infections usually occur when *B. fragilis* escapes from the gastrointestinal tract to other areas of the body due to gut damage (rupture of an inflamed appendix, surgery, trauma, diverticulitis, postpartum endometritis, malignancy, etc.) [6,112]. ETBF strains can lead to abscess formation in multiple sites of the body (including the liver, lung, and brain) and potentially extraintestinal complications, such as central nervous system complications, cancer, endocarditis, bacteremia, and septicemia [1,6]. In fact, the BFT has been demonstrated to be toxic in vitro to kidney and lung epithelial cells, as well as to endothelium, suggesting its potential role in extraintestinal infections [32,113], although the implication of the BFT in extraintestinal infections and abscess formation has not yet been clearly defined.

There is evidence that *B. fragilis* might have a pathological role in neurodegeneration through the BFT and circulating metabolites [114]. Surface lipopolysaccharides (LPSs) of *B. fragilis* have been found to be potent inducers of the pro-inflammatory transcription factor NF-kB (p50/p65 complex) in human co-cultures of brain cells, a trigger in the expression of pathogenic pathways involved in inflammatory neurodegeneration [115].

Besides host genetic factors, an imbalance in the normal gut microbiota can promote chronic inflammation and carcinogenic metabolite production, leading to neoplasia [116]. *B. fragilis* has received particular attention for a possible association and role in the initiation as well as the progression of CRC. Particularly, there is evidence that several cellular changes associated with CRC pathogenesis (morphological/proliferation changes, changes in cell permeability/cytotoxicity response, and gene expression changes) can be induced by exposure to the BFT, ultimately leading to tissue damage and tumorigenesis [117].

#### 3.2.3. Antibiotic Resistance of *B. fragilis*

*B. fragilis* has been found to have higher levels of antibiotic resistance and numerous arrays of antibiotic resistance mechanisms compared with other anaerobic bacteria in the gut [118,119]. A decrease in susceptibility levels for several antibiotics, including β-lactams, tetracyclines, and macrolides, besides the emergence of multidrug-resistant (MDR) strains, complicates *B. fragilis* infection treatment, especially in patients with a history of exposure to antibiotics [49].

*B. fragilis* has a plastic genome that facilitates the accumulation of antibiotic resistance determinants [120]. Resistance to penicillin and ampicillin in this bacterium is mediated by chromosomal β-lactamases (*cfiA* and *cepA* genes) [121], while resistance to macrolide-lincosamide-streptogramin B is usually associated with *erm* genes, usually *ermF* but also *ermB* and *ermG*, that have been observed on both plasmids and conjugative elements [122]. Resistance of *B. fragilis* to tetracycline is inducible and related to the ribosomal protection of TetQ [123], whose gene is located on large transferable transposons [122]. Differently, efflux mediated mechanisms, along with amino acid substitution of topoisomerases, in particular Ser82 → Phe in the gyrase A (Gyr A), are associated with fluoroquinolone resistance in this bacterium [123].

Carbapenems and metronidazole are key drugs for prophylaxis and the empirical treatment of sepsis, particularly in the polymicrobial intra-abdominal and soft tissue infections [124,125]. However, an increased resistance to these antibiotics among clinically isolated and commensal *B. fragilis* strains has recently been reported [124,125].

Resistance to carbapenems in *B. fragilis* is associated with the production of metallo-β-lactamase, characteristically encoded by the chromosomal *cfiA* gene, whose expression is activated by the presence of an insertion sequence (IS) element located upstream of the gene [126]. Since some resistant *B. fragilis* strains do not carry the IS element upstream of the *cfiA*-coding region but carbapenem resistance has been reported in *cfiA*-negative isolates, Ogane et al. have hypothesized that resistance may also be due to the accumulation of mutations in outer membrane porins and penicillin-binding proteins [118]. The MICs of isolates with the *cfiA* gene for imipenem have been reported lower than those for doripenem and meropenem, so imipenem has been considered to be preferred for an empirical treatment of *B. fragilis* infections [118].

Recently, treatment failures and complications due to *B. fragilis* strains resistant to metronidazole (MTZ) have increased [125]. MTZ resistance is usually coupled with *nim* genes, which can be either chromosomal or plasmid-borne, are frequently associated with IS, and have been reported as transferable by a conjugative process [125]. These genes have been found in *B. fragilis* strains susceptible to MTZ, in which they are probably expressed at a very low level or not expressed at all [125]. *B. fragilis* strains highly resistant to MTZ and negative for *nim* genes have also been isolated, suggesting additional mechanisms of resistance. Steffens et al. have reported that overexpression of the RecA, a major DNA repair protein in *B. fragilis*, can lead to increased resistance to UV radiation and MTZ treatment [127]. Furthermore, efflux pumps also contribute to MTZ resistance, and resistance-nodulation-division pump system (RND)-type multi-drug efflux pumps, such as the BmeRABC5 system, have been identified in *B. fragilis* strains with increased MICs to this antibiotic [128]. *B. fragilis* susceptibility to MTZ is also linked to bacterium iron status. A deficiency in the FeoAB ferrous iron transporter reduced intracellular iron uptake, subsequently impairing the activation of MTZ and leading to increased resistance [129]. Although this mechanism has not been associated with clinical resistance, it indicates that iron homeostasis is crucial for the intracellular activation of MTZ. Finally, facultative anaerobic organisms, such as *Enterococcus faecalis*, can protect obligated anaerobes like *B. fragilis* from MTZ by providing an environment that promotes anaerobe growth and by inactivating the drug [130].

The emergence of *B. fragilis* MDR strains has been registered in several countries, potentially strongly limiting the options of treatment for the associated infections, since MDR strains can show resistance to the antibiotics used to treat anaerobic infections [131]. In addition, recent data indicate that antibiotic resistance and resistance genes have been found not only in ETBF but also in NTBF strains [119,132,133], indicating that NTBF may represent an important reservoir for antibiotic resistance determinants.

*B. fragilis* antibiotic resistance appears a complex and multifactorial phenomenon, and a more complete understanding is necessary for better control and treatment of *B. fragilis* diseases. Furthermore, antibiotic therapy should be implemented based on the clinical evaluation and the antibiotic susceptibility analysis, considering that *B. fragilis* infections, especially intra-abdominal infections, often show a polymicrobial nature that requires therapy with a broad spectrum of effects on both anaerobic and facultative anaerobic bacteria.

Although *B. fragilis* is one of the most clinically relevant species, data collection is complicated, since this bacterium is not included in the major public surveillance systems, and most studies combine data of several anaerobic bacteria from several clinical sources, only a minority of which includes data differentiating between species and clinical samples. Differentiated resistance profiles for the different species should be important, considering that resistance mechanisms within the genus *Bacteroides* have been found to be species-dependent [134].

## 4. *B. fragilis* Interaction with *C. difficile*

*B. fragilis* exhibits complex interactions with the other bacteria, affecting the health of the human host. For example, a study using a polymicrobial infection model of intra-abdominal abscess formation, has reported a synergistic relationship between *B. fragilis* and *Escherichia coli* with an increase in the abscess weight and in the abundance of *E. coli* after the addition of *B. fragilis* [135]. Other authors, using animal models, have demonstrated that *B. fragilis* can prevent the experimental colitis induced by *Bartonella henselae* and *Helicobacter hepaticus* through the PSA, which exerts its effects within both the intestinal and the systemic compartments [136].

*B. fragilis* interaction with *Clostridioides difficile* (CD) appears intriguing, showing multifaceted aspects. Recent data suggest a *B. fragilis* role in preventing *Clostridioides difficile* infection (CDI) [24,137,138], but also that both *B. fragilis* and CD can contribute to CRC development and progression, exerting a tumor-promoting effect and participating in constituting and influencing the tumor microenvironment (TME) [26,139].

### 4.1. Clostridioides Difficile Infection (CDI)

CD is an anaerobic, Gram-positive spore-forming bacillus, known as a major nosocomial pathogen causing antibiotic-associated diarrhea and pseudomembranous colitis cases [140]. Asymptomatic carriage of toxigenic CD strains is less than 5% in adults from the community but rises to 11% and 19% in patients in hospitals and in the long-term care facilities, respectively [141,142]. A healthy gut microbiota provides natural immunity to CDI, while a disturbed gut microbiota and the consequent loss of colonization resistance, usually after antibiotic treatment, are associated with this infection [143,144]. Gut alterations lead to a decrease in beneficial bacteria, such as *Bacteroides*, *Prevotella*, *Enterococcaceae* spp, *Leuconostocaceae* spp and *Bifidobacterium*, with an in increase in the abundance of *Aerococcaceae*, *Enterobacteriaceae*, *Clostridioides* and *Lactobacillus* positively correlated to CD colonization and CDI development [145,146].

Bile acids and salts have a considerable impact on CD colonization of the human gut. While primary bile acids, such as the taurocholic acid, produced by gut microbiota are required for CD germination, secondary bile acids can inhibit CD growth [147,148,149]. However, the production of secondary bile acid appears to be only part of the colonization resistance mechanism. Hassall et al. have demonstrated in vitro that CD growth is substantially impacted by gut microbiota biofilms, the abundance of commensal bacteria, including *Escherichia coli, Proteobacteria, and Bacteroides* spp, high levels of secreted inhibitory products, and competition for space and nutrients [137].

**Figure 1:**
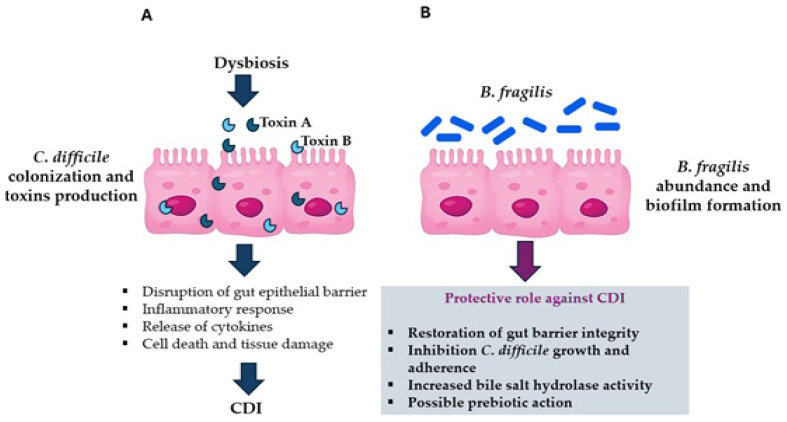
Schematic representation of *C. difficile* infection (CDI) pathogenesis (**A**) and *B. fragilis* protective role against CDI (**B**).

CDI usually occurs when the gut microbiota is altered by antibiotic treatment, particularly by treatment with cephalosporins, amoxicillin/clavulanic acid, carbapenems, fluoroquinolones, and clindamycin [145,150]. Besides antibiotic use, the main risk factors for CDI also include age over 65 years, impaired immune status and co-morbidities, use of proton pump inhibitors (responsible for intestinal dysbiosis) and recent/prolonged hospitalization [151]. CD virulence is mainly due to two toxins, toxin A (TcdA) and toxin B (TcdB), encoded by the *tcdA* and *tcdB* genes, respectively, that are part of the 19.6 kb genetic pathogenicity locus (PaLoc), as well as the *tcdD,* the *tcdE*, and the *tcdC* genes that encode for regulatory proteins [152,153,154]. CD toxins act on the actin skeleton and disrupt epithelial barrier integrity, causing inflammation of the colon, cell death and tissue damage [155] (Figure 1).

Certain strains of CD, including the global epidemic strains recognized as ribotype (RT) 027 and RT 078 by the PCR ribotyping method, produce a third toxin, the binary toxin (CDT), that might increase their virulence, acting in synergy with toxins A and B in the destruction of the actin cytoskeleton and intestinal epithelial cell apoptosis [156].

Since the 2000s, the epidemiology of CDI has dramatically changed, and severe CDI outbreaks have been reported in North America and Europe due to the CD epidemic RT 027 and, successively, by other CD types characterized by high virulence that have more recently emerged [157,158]. CD has been recognized as an emerging pathogen in the community in recent years, with increased cases reported worldwide [159].

As an obligate anaerobe, vegetative CD cannot survive in an atmosphere containing oxygen, while CD spores can survive and persist in hostile environments [151]. When ingested by the fecal-oral route and reaching the duodenum, they stick to intestinal cells and germinate to vegetative cells [160]. Germination of CD spores occurred in response to chemical signals (germinants), primarily bile acids and mainly taurocholate, which induce the spores to revert into vegetative cells [161]. CD spores are a major cause of persistent infection and recurrences because they can survive on surfaces for a long time and are highly resistant to antibiotics and disinfectants [162]. After CDI, asymptomatic patients may continue to shed CD spores in their feces for up to four weeks or more, re-contaminating hospital environments and acting as a source for new infections or recurrences [162]. CDI recurrences (rCDI) occurred in 20% to 30% of CDI patients, with an increased percentage of mortality, also because, after a first recurrence, patients often experience subsequent recurrences that are difficult to treat and that increase the burden for healthcare facilities [163]. A healthy gut barrier and microbiota are necessary to resist CD colonization and infection. For these reasons, restoring a healthy gut microbiota net through fecal microbiota transplantation (FMT) is now recommended by international guidelines as excellent therapy for rCDI and preventing its complications [164].

Paradoxically, antibiotic treatment is the first primary risk factor for CDI, but it is also the first-line therapy for this infection. Currently, vancomycin (VAN) and fidaxomicin (FDX) are the primary antibiotics for CDI treatment, while MTZ is not yet suggested as a first-line antibiotic due to the high percentage of associated treatment failures and recurrences [165]. Other novel narrow-spectrum antimicrobials are also in development for CDI treatment, such as surotomycin, cadazolid, and ridinilazole [166]. A plethora of antibiotic resistance mechanisms have been found in CD, including acquisition of mobile genetic elements, antibiotic target mutations, changes in the expression of redox-active proteins, biofilm production, etc. [131]. Furthermore, genetic elements can move within and between CD strains and other bacterial species, amplifying their diffusion [167]. In addition, the percentage of CD strains showing multiple antibiotic resistance (MDR) is increasing, particularly among epidemic strains, and the MDR patterns are often involved in resistance to fluoroquinolones, MLSb, and rifampin [131].

### 4.2. B. fragilis Protective Role Against CDI

Several findings support a *B. fragilis* protective role against CDI. Goldberg et al., in a study including 59 patients with CDI and without CDI, observed an inverse association between CDI and the abundance of *B. fragilis*, supporting that it can be involved in CDI protection [168] (Figure 1). Experiments with animal models have indicated that *B. fragilis* strain ZY-312, isolated from a healthy infant, has shown protective effects against CDI in rats, regulating microbiome and restoring gut barrier integrity [169]. Furthermore, in another study, prophylactic colonization of pathogen-free mouses with a single strain of *B. fragilis* (*B. fragilis* 3_1_12) has been shown to decrease CD adherence, reduce CDI-induced transepithelial electrical resistance and attenuate cells apoptosis [137].

During infection, CD biofilm allows this pathogen to persist in the gut also in the presence of antibiotics, with a potential role in reestablishing infection with recurrences [170]. Interestingly, co-culturing CD with *B*. *fragilis* in mixed biofilms reduces CD growth, and this inhibition seems to be linked to the potent cross-species quorum sensing group of molecules CD LuxS/AI-2, which may induce selective metabolic responses in *B. fragilis* ultimately hindering CD growth [171,172].

*B. fragilis*, like other *Bacteroides* species, produces bile salt hydrolases that deconjugate bile acids, a step necessary to process primary bile acids into secondary bile acids [173]. The conjugated primary bile taurocholic acid acts as a strong germinant of CD spores, while the secondary bile deoxycholic acid acts as an inhibitor of CD growth [148,174,175]. Interestingly, it has been observed that bile salt hydrolase activity is increased in patients subjected to FMT that are subsequently protected from CDI recurrences [176]. Several bacterial species are important for the successful outcome of FMT, including *Bacteroides fragilis*, *Roseburia intestinalis*, and *Faecalibacterium prausnitzii* [177]. A study on six patients with CDI that received FMT has reported that the levels of *Bacteroides,* including *B. fragilis,* increased in FMT recipients, besides an increase in secondary bile acids deoxycholate, lithocholate, and ursodeoxycholate [178].

In a recent study, Imwattana et al. have found that *B. fragilis* clinical isolates collected in Thailand are resistant to antibiotics associated with increased CDI risk, such as clindamycin [179]. In this study, preliminary in vitro experiments have demonstrated that clindamycin-resistant *B. fragilis* strains maintain their ability to inhibit CD growth in the presence of this antibiotic, while the inhibitory effects of clindamycin-susceptible strains diminish under the same conditions, suggesting that resistance to antibiotics in *B. fragilis*, combined with the ability to limit CD adhesion to the gut mucosal surface, may play a role in preventing CDI.

Some data suggest *B. fragilis* as a prebiotic agent for the CDI treatment. A very recent study reports that the presence of the *B. fragilis* strain ZY-312 in the gut can lead to an increase in the beneficial *Akkermansia muciniphila*, which prevents CD colonization [180] (Figure 1). Dietary supplementation of fructooligosaccharides (FOS) and soy fiber has been shown to delay CDI onset, attenuate CDI development, and increase survival time in hamsters [181,182]. In addition, Lewis et al. found that hospitalized patients with CDI who received oligofructose, a fructooligosaccharide (FOS), were less likely to develop recurrent CDI, although they continued to have positive cultures for CD at 30 and 60 days, reflecting a persistent colonization [182]. Furthermore, Piotrowski et al. has reported that NTBF can reduce CD biofilm formation when co-incubated in vitro and that a co-culture of CD with NTBF and 1% FOS can inhibit CD adhesion to cells [183]. Although further investigations and specific studies are necessary, these results indicate that CD metabolism and the pathophysiology of CDI involve complex interactions between diet, intestinal bacteria, and environment, with potentially important impacts on prevention and treatment of CDI.

### 4.3. Involvement of B. fragilis and C. difficile in Colorectal Cancer (CRC)

Colorectal cancer (CRC) is a major global health concern, with an estimated 1.9 million new cases and 0.9 million deaths in 2020 [184,185]. The development of CRC is a complex process influenced by genetic, environmental, and lifestyle factors, including intestinal bacteria, revealing strong associations between specific bacteria, the TME, and cancer pathways [186,187].

Two main pathways lead to CRC development: the adenoma-carcinoma pathway and the serrated pathway [188]. The adenoma-carcinoma pathway involves early mutations in the tumor suppressor gene adenomatous polyposis coli (APC) and subsequent chromosomal instability (CIN) [189]. The serrated pathway, accounting for about 15% of all sporadic CRC cases, originates from sessile serrated adenomas [190,191].

Large-scale sequencing technologies have contributed to the development of a Consensus Molecular Subtyping (CMS) system for CRC based on tumor gene expression. Four CMS subtypes have been identified among the two pathways of carcinogenesis: CMS1 relates to the serrated pathway, and CMS2, CMS3 and CMS4 relate to subtypes of the CIN pathway [192].

Interestingly, recent analysis of differences in taxonomic abundances has shown changes at the phylum level in the gut microbiota, indicating a strong association of CMS subtypes with *B. fragilis* and CD, which are involved in various aspects of CRC carcinogenesis [188,192,193].

#### 4.3.1. *B. fragilis* Role in CRC

*B. fragilis* has received significant attention for a possible association with CRC and a causal role in carcinogenesis [1]. There is evidence suggesting an ETBF role in enhancing tumorigenesis, facilitating growth and metastasis of CRC cells, and damaging the intestinal mucosal barrier. Differently, there is contradictory data on the NTBF impact on CRC cell proliferation or metastasis, so further investigations are needed to clarify the role of NTBF in CRC.

Interestingly, a recent study using whole genome sequencing (WGS) on one ETBF strain (bft1-producing ZY0302) and one NTBF strain (ZY0804), isolated from cancerous and paraneoplastic tissues, respectively, has found that the TME can exert pressure selection, potentially driving pan-genomic variability, with alterations in both core and nonessential genes along with significant instances of horizontal gene transfer, and facilitating *B. fragilis* strains adaptation to the changing TME [194].

##### Enterotoxigenic *B. fragilis* (ETBF)

Several studies have reported ETBF strains significantly increased in both patients with cancerous and precancerous lesions compared to patients with healthy mucosa, suggesting that these strains can represent possible drivers in CRC [25,195,196,197,198].

**Figure 2:**
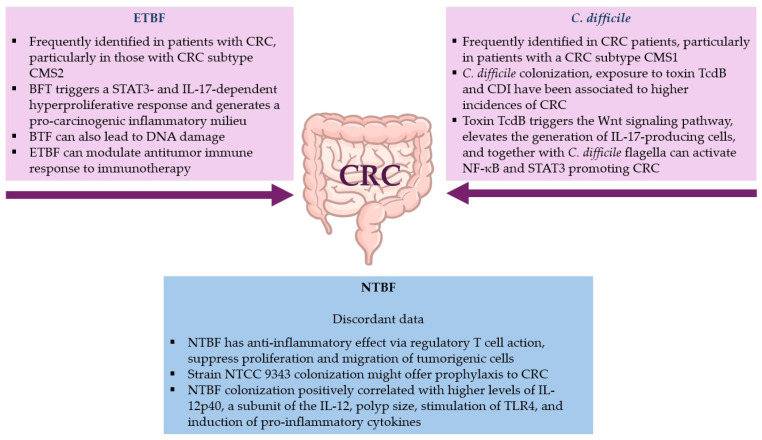
Enterotoxigenic *B. fragilis* (ETBF), non-toxigenic *B. fragilis* (NTBF) and CD contribution to colorectal cancer (CRC).

Although a low bacterial biomass has been associated with the CMS2 subtype of CRC, a higher proportion of *B. fragilis* has been observed in patients with tumors of this subtype compared to patients with other CMS subtypes [192] (Figure 2).

Interestingly, an increased abundance of *B. fragilis* has been associated with a high-fat diet with a high consumption of red meat that stimulates the bile flow and, in turn, *B. fragilis* to convert bile to metabolites and fecapentaenes, considered to be cocarcinogens or mutagens, contributing to increasing the risk of CRC [199,200].

ETBF strains have been associated with persistent colitis, epithelial barrier disruption, and inflammation, potentially promoting CRC [55,201,202]. Furthermore, a study on multiple intestinal neoplasia (Min) mice, which are genetically predisposed to intestinal tumors, has shown that mice spontaneously develop tumors after two–three months in the presence of their endogenous microbiota, while tumors appear about four weeks after oral administration of ETBF [203].

The upregulation of IL-8, increasing cellular proliferation, angiogenesis, and cell migration, is known to promote CRC [204]. Notably, it has been observed that the expression of the IL-8 gene (CXCL8) increases fivefold when CRC cell lines are co-cultured with ETBF strains [205].

Experiments in animal models have demonstrated that the BFT is involved in tumorigenesis through the stimulation of T helper cells type 17 (Th17), which leads to colonic epithelium damage by the IL-17 release, and the activation of the signal transducer and activator of transcription 3 (STAT3) pathway, which triggers a hyperproliferative response and generates a pro-carcinogenic inflammatory environment [203,205,206,207,208]. Interestingly, all BFT variants induce acute IL-17–dominant colitis and studies on ETBF with *bft2* have shown that colonization with IL-17–dominant colitis persisting for up to one year in C57BL/6 mice [209].

The lack of the functionality in the APC gene, a crucial tumor suppressor, seems to be the main driver of early-onset CRC, in fact variations in APC gene have been reported in 70–80% of both sporadic and familial CRC cases [46,208]. A non-functional APC and the effect of the BFT on E-cadherin lead to a free cytosolic pool of β-catenin, activating oncogenic transcription, specifically upregulating c-Myc, and promoting cellular hyperproliferation and crypt hyperplasia [50,210,211,212,213]. Recent findings also indicate that the BFT can promote proliferation of tumorigenic cells through the downregulation of miR-149-3p, which regulates differentiation of Th17 cells [13].

Several studies highlight the role of the BFT in DNA damage, DNA methylation, and chromatin accessibility changes [209,212,214] (Figure 2). In fact, the BFT can induce the phosphorylation of H2AX (γ-H2AX), a marker of DNA damage, and stimulate the production of reactive oxygen species (ROS) through spermine oxidase (SMO) [215]. A recent study, using whole-genome and whole-exome sequencing in *Apc*^min/+^ and *Apc*^min/+^*Msh2^fl/fl^* VC mice to determine if ETBF induces mutations that can impact the *Apc* gene, other tumor suppressors, or proto-oncogenes, shows that ETBF does not produce a unique mutational profile and that ETBF-induced tumors arise from errors in DNA mismatch repair and homologous recombination DNA damage repair [216].

*B. fragilis* appears to play a role in both chemotherapy resistance and the modulation of the antitumor immune response to immunotherapy (Figure 2). Very recently, Ding et al. have conducted a cross-sectional comparison of gut microbiomes of responders and non-responders to chemotherapy in two independent CRC cohorts [217]. The study has indicated that *B. fragilis* is abundant in non-responders and associated with poor prognosis, suggesting that this bacterium can have a role in driving chemoresistance. The mechanism involved the *B. fragilis* surface protein SusD/RagB, which, binding to the Notch1 receptor in CRC cells, leads to activation of the Notch1 signaling pathway, inducing epithelial-to-mesenchymal transition (EMT)/stemness to suppress chemotherapy-induced apoptosis. Vétizou et al. have reported that mouse tumors, grown in germ-free conditions or in the presence of broad-spectrum antibiotics, are unresponsive to cytotoxic T-lymphocyte-associated antigen 4 (CTLA-4) [218]. When mice are recolonized with *B. fragilis*, tumors regained responsiveness to CTLA-4 treatment, with an elevated T helper 1 (Th1) response and maturation of intratumorally dendritic cells (DCs), suggesting a role of *B. fragilis* in bolstering immunotherapy.

##### Non-Toxigenic *B. fragilis* (NTBF)

NTBF strains are generally considered to have an anti-inflammatory effect via regulatory T cell action [206]. Sittipo et al., using a CRC cell line, have reported that the NTBF-derived PSA from *B. fragilis* NCTC 9343 can regulate the cell cycle via TLR2 signaling and the epithelial–mesenchymal transition, suppressing both tumorigenic cells proliferation and migration [219] (Figure 2). Notably, Lee et al. have found that *B. fragilis* NCTC 9343 has a protective role against CRC in the AOM/DSS model, and they have also observed an inhibited expression of C-C chemokine receptor 5 (CCR5) in the gut besides a decreased carcinogenesis after NTBF administration [220].

Chan et al. have observed that in both SPF C57BL/6 wild-type (WT) and multiple intestinal neoplasia (MinApc716^+/−^) mice the sequential treatment with the NTBF strain NTCC 9343 followed by the ETBF strain 86-5443-2-2 diminishes colitis and tumorigenesis [12]. Differently, when mice are simultaneously treated with both the NTBF strain NTCC 9343 and the ETBF strain 86-5443-2-2 severe colitis and tumorigenesis are observed. Reduced severity of disease in mice sequentially treated has been attributed to the prevalence of NTBF strain NTCC 9343 and the consequent decrease in IL-17A. The authors have also observed that although gut colonization by ETBF can abrogate the anti-inflammatory effect of NTBF, it may not be effective in treating colitis once it is established [12].

Differently, Yang et al., using both ETBF and NTBF strains (NCC336948; BNCC) in SW480 cells and a Caco2 intestinal barrier models, have observed that NTBF does not significantly inhibit CRC cell malignancy in vitro, although it might have a role in preventing damage to the intestinal mucosa [221]. In addition, a study by Kordahi et al. suggests a possible role of NTBF strains in the early stages of CRC development, reporting that the NTBF isolates from patients with polyps are significantly enriched in LPS biosynthesis genes, positively correlated with higher levels of IL-12p40, larger polyp size, and stimulation of TLR4, which leads to the induction of pro-inflammatory cytokines [222].

Although NTBF has been recognized as a potential next-generation probiotics (NGPs) candidate, these data, with other recent studies, indicate that NTBF may potentially worsen conditions such as CRC, diabetes, and atherosclerosis [221,222,223], and that the beneficial effects of NTBF are strain-specific. Further investigations are needed to fully understand NTBF’s intricate relationship with the host and identify specific factors that determine whether an NTBF strain may have beneficial or detrimental effects.

#### 4.3.2. *C. difficile’s* Role in CRC

Recent investigations suggest a role of CD in CRC development, highlighting the important effects of associated gut microbiota dysbiosis and pathogenic effects of CD flagella and toxins [26] (Figure 2).

Fecal samples from CRC patients were found to contain several *Clostridium* species with flagella, with a greater abundance of CD compared to healthy mucosa [224,225]. CD has frequently been identified in CRC patients, with a percentage up to 80%, suggesting a possible involvement of this pathogen in CRC [226,227,228,229,230,231].

In a longitudinal study, Geier et al. have found a 2.7-fold increase in the CRC incidence in patients with a CDI diagnosis compared to those without CDI [232]. Furthermore, they have also observed that rCDI is more frequently associated with a higher CRC incidence. Differently, a recent study by Patel et al. has reported a decrease in the CRC incidence in patients with a history of CDI compared to healthy patients [233]. However, these authors have also observed that the presence of both obesity and a previous CDI significantly increase the risk of CRC. Therefore, obesity could have an additive effect with CDI in exacerbating gut inflammation and increasing the risk of malignancy [233]. Notably, rates of CD colonization have been found to be significantly higher in patients with lymph node metastasis compared to others without lymph node involvement, also suggesting a possible role of this pathogen in cancer metastasis [226].

Recent evidence indicates that prolonged CD colonization with exposure to TcdB, but not to TcdA, seems necessary for induction of CRC, although not all CD strain seem to possess pro-carcinogenic properties [234]. Furthermore, antibodies against TcdA and TcdB seem to prevent CDI but not to protect against CD colonization [235]. Persistence of mucosal CD colonization, abundance of TcdB production, and impact of differing CD types represent critical gaps that need to be filled to clarify the role of CD in CRC.

CDIs are usually associated with an altered gut microbiota, a condition known as dysbiosis [236,237]. Dysbiosis causes the loss of beneficial microbiota, producing anti-inflammatory metabolites, such as short-chain fatty acids (SCFAs) that include acetate, propionate, and butyrate [238], a reduction that can exacerbate the pathological effects of CDI, promoting pathogenic bacteria growth and aggravating inflammation and epithelial stress. CDI and all these associated microbial changes can trigger tumorigenesis. Using mouse *APC*^min+^ models, it has been observed that CD has tumorigenic potential and that chronic CDI can accelerate tumor growth; furthermore, *APC*^min+^ mice infected with mutated TcdB CD strains developed fewer tumors [26,187]. Drewes et al. have observed that in mouse *APC*^min+^ models, CD toxin TcdB triggers the Wnt signaling pathway and elevates the generation of IL-17-producing cells, supporting the role of this toxin in tumorigenesis [187].

Bacterial flagella are recognized by the innate immune system via toll-like receptors (TLR5) that induce the myeloid differentiation primary-response gene 88 (MyD88) activation, leading to pro-inflammatory chemokine and cytokine release [239]. Subsequently, various immune cells, including dendritic cells, neutrophils, and monocytes, are recruited and activated, inducing further inflammatory cytokine production [240]. The adaptive immune system is activated by dendritic cells, with recruitment of B and T cells and then neutralization and elimination of pathogens within the TME [241].

CD flagella seem to have a functional role in CRC carcinogenesis towards a CMS1 subtype. In mouse models, CD flagella induce the activation of MAPKs and the nuclear factor-κB (NF-κB) and the secretion of pro-inflammatory cytokines, including IL-6 and IL-1β, through TLR5 signaling [242], contributing to a pro-inflammatory TME and increasing immune infiltration, as observed in CRC subtype CMS1.

Interestingly, CD toxins besides NF-κB can also activate the STAT3, another inflammatory signaling pathways [243,244], with a large production of pro-inflammatory cytokines and chemokines (such as IL-6 and IL-8), and the tumor necrosis factor-alpha (TNF-α), that ensure the survival of damaged cells and accelerate mutated cells proliferation, creating a tumorigenic environment [245]. Activation of the STAT3 prevents programmed cell death, contributing to the survival of precancerous cells by increased expression of anti-apoptotic genes, such as those for the myeloid cell leukemia 1 (Mcl-1) and B-cell lymphoma 2 (Bcl-2) [246,247].

Future investigations on possible epidemiological links between CD and CRC, as well as on factors that facilitate persistent CD colonization and production of toxins in the human gut, will be necessary to better understand the oncogenic potential of this pathogen.

## 5. Conclusions and Future Research

The expansive pan genome of *B. fragilis*, characterized by a large genetic diversity, contributes to both its adaptability and pathogenic potential, allowing this bacterium to act as an integral partner in the human metabolic system but also as an opportunistic pathogen, causing infections and promoting important human diseases.

Besides the production of the toxin BFT, the main virulence factor that distinguishes ETBF from NTBF strains, *B. fragilis* also shows reversibly variable expression of components, such as surface structures, that lead to variable physiological and biochemical characteristics within a single strain, facilitating adaptation and evasion from the immune system of the host. Extensive genomic comparisons by high-throughput sequencing technology could help to clarify how genomic diversity drives *B. fragilis* strains adaptation and differentiation and identify which genetic traits distinguish and differentiate ETBF strains, associated with infections and diseases, from beneficial commensal NTBF strains.

The capability to mitigate inflammation, to prevent pathogen colonization, and to modulate the immunological response, with beneficial effects for the host, makes NTBF a promising candidate as a NGP, although further investigations are needed to understand how these properties are linked to the different NTBF strains, how these strains interact within diverse gut environments, and their long-term impacts on human health and disease. In addition, some NTBF strains can elude the human host’s defenses and migrate from intestinal tissue, causing abscesses and contributing to the progress of some human diseases, and NTBF strains also have the capability of accepting and spreading antibiotic resistance determinants and virulence factors, posing significant challenges in their use as NGP [248,249]. For these reasons, the evaluation of the safety and efficacy of *B. fragilis* as an NGP requires several steps, including in vitro and in vivo experiments. Besides WGS, integrating multi-omics and advanced bioinformatics (including artificial intelligence (AI) and machine learning (ML) technologies) that analyze data from in vitro, in vivo, and in silico appear critical to determine the genomic composition and precisely characterize and categorize NGP candidates [250,251]. Considering that the gut microbiota is variable among the different individuals, stemming from factors like diet and genetics, engineered NGPs can enable researchers to develop personalized therapies that target specific pathways based on an individual’s distinct microbiome profile, optimizing therapeutic outcomes. Interestingly, genetic engineering also offers promising solutions to enhance *B. fragilis* NGPs functionality, stability, viability, and health benefits. For example, using CRISPR-Cas systems, NGPs can be engineered to carry plasmid vaccines that prevent the spread of antibiotic resistance genes among gut microbiota or, using advanced coating strategies, such as nanoarmor, to enhance their stability and mucoadhesive capacity [62]. However, while NTBF NGPs have been demonstrated to reduce inflammation and improve gut barrier function in animal models, the transition to human studies is still in its early stages, with a shortage of human clinical trials that replicated and proved safe and effective NTBF NGPs in humans before regulatory approval and application.

The dual nature of *B. fragilis* poses challenges for some clinical interventions, such as the FMT that aims to restore healthy gut microbiota. The accidental transplanting of *B. fragilis* strains with pathogenic potential through FMT could disrupt the gut microbiota’s delicate balance, potentially causing disease instead of health benefits. To address this risk, it is crucial to identify and characterize beneficial *B. fragilis* strains while simultaneously understanding their complex interactions with the host and gut bacteria at a molecular level. These critical needs are also highlighted by the recent emergence of MDR *B. fragilis* strains and the alert by the USA Food and Drug Administration (FDA) about MDR microorganisms as a potential risk related to FMT [252]. In this regard, it is interesting to note that the transplantation of the human gut microbiota into gnotobiotic animals, combined with statistical modeling, has recently been proposed as a promising approach to evaluate FMT efficacy, select optimal donors, and identify key bacterial strains for future therapeutic use, such as NGP candidates [253].

Interestingly, *B. fragilis* produces bile salt hydrolases that increase the level of secondary bile acids, inhibitors of CD growth. Additionally, the presence of *B. fragilis* in the gut seems to promote CD-protective bacteria, such as *Akkermansia muciniphila*, and impact CD biofilm formation. However, further research on the molecular mechanisms that underlie the *B. fragilis* inhibition of CD colonization is needed to fully explain *B. fragilis* protective effect and to develop *B. fragilis* NGPs to prevent CDI and its recurrence. In fact, as a potential NGP or in association with FOS [183], *B. fragilis* could offer an alternative approach to CDI prevention and treatment, but its use must be carefully evaluated since both these bacteria play a role in the development of CRC in humans. There is also a limited understanding of specific *B. fragilis* interactions not only with CD but also with the host and the other bacterial species, which may influence the overall gut microbiome structure, stability, and function and impact the health and susceptibility to diseases of the human host, highlighting the pressing necessity for further investigations to better understand these intricate relationships, especially in CRC development.

Both *B. fragilis* and CD contribute to colorectal cancer (CRC) along specific pathways and CMS subtypes. ETBF has been associated with increased inflammation, genomic alterations, increased tumor cell proliferation and migration, and altered host immune response, although many aspects of its contribution to CRC remain unclear and need to be explored. Differently, there is still contrasting data on the role of NTBF, which has been demonstrated to show an anti-inflammatory effect and anti-tumorigenic effects but also to positively correlate with higher levels of pro-inflammatory cytokines and larger polyp size, without a significant capability to inhibit CRC cell malignancy in vitro. CDI causes epithelial barrier disruption, DNA damage, and chronic inflammation. Furthermore, CDI is associated with dysbiosis that exacerbates tumorigenesis by altering gut microbiota and microbial metabolites.

A recent analysis of data has led to a hypothetical scheme of bacteria cooperation in developing CRC, in which during the precancerous stage of CRC, *B. fragilis* BFT causes inflammation and mucosal damage that allow pks+ *E. coli* colonization, the induction of genetic mutations in the carcinogenesis stage, and the recruitment of *Fusobacterium nucleatum* to colonize the lesion site and promote stemness and proliferation of cancer cells, contributing to CRC advancement [254].

Interestingly, commonalities can be observed between *B. fragilis* and CD mechanisms in CRC development. In fact, both BFT and CD trigger mutated cells hyperproliferation and contribute to a pro-inflammatory TME, stimulating the production of IL-17 and activation of the STAT3 pathway, thus promoting colon carcinogenesis. A CD persistent colonization of the gut, besides the production of toxins, seems to be necessary for CRC induction [234]. However, the persistence of CD in feces and mucosa, the conditions for the occurrence of a persistent CD colonization (including the possible impact of the different CD types and the immunological response of the host), the link between inflammation and a persistent colonization by CD, and the duration of CD toxin production in the colon remain unclear factors in the oncogenic potential of this bacterium.

Remarkably, both *B. fragilis* and CD show a high percentage of MDR resistance, and MDR has an impact on CRC development [255]. In fact, inappropriate and repeated antibiotic exposure leads to dysbiosis and drives acquisition and spread of antibiotic resistance among gut bacteria, rising pro-inflammatory bacteria, and increasing the necessity of further antibiotic treatments and, finally, the risk of CRC. This scenario emphasizes the need for more investigations into the impact of the different antibiotics and doses on the gut microbiota, particularly on *B. fragilis* and *C. difficile*, in relation to CRC development. Furthermore, multi-omics approaches could also have an important impact in identifying genes and pathways that are involved in antibiotic resistance, tracking the geographical and temporal spread of resistance determinants, recognizing the selective pressures and evolutionary trends influencing *B. fragilis* resistance, and developing targeted treatments that can overcome resistance and effectively manage *B. fragilis* infections.

Recent advances in understanding gut microbiota interplay with the human immune system may offer new insights into preventing and managing CRC, although the clinical application is complicated by host genetics and environmental factors. Integrating multi-omics data could be of help in CRC patient management and in the identification/characterization of bacterial strains with anticancer properties, like certain NTBF strains, and pathogenic bacteria involved in CRC, such as ETBF, that could be targeted by new vaccines.

In conclusion, *B. fragilis,* with its peculiar characteristics, has an ambivalent role in the health of humans that complicates its potential use as a probiotic or NGP, as well as in clinical intervention such as FMT. *B. fragilis* complex interactions with the other intestinal bacteria and its role in the development but also in the prevention of CRC represent a critical area for future research that, using multi-omics approaches, along with genomic analyses of strain diversity and the study of virulence factors, can clarify *B. fragilis* physiological responses to environmental challenges, its interactions with the host immune system and the other gut bacteria, and the impact of genetic variation on its functional and pathogenic potential.

## Figures and Tables

**Table 1:** Commensal and pathogenic characteristics of non-toxigenic *B. fragilis* (NTBF) and enterotoxigenic *B. fragilis* (ETBF).

**Commensal Role**	
	**NTBF and ETBF strains**
	Degrade polysaccharides of plants
	Produce short-chain fatty acids
	Show regulation properties
	Show anti-inflammatory properties
	Prevent gut dysbiosis
	Prevent bacterial infection
	Mitigate several diseases
	**ETBF strains**
	Abolish *B. fragilis* toxin (BFT) production or produce a nondamaging BFT in the intestine of carriers, favoring bacterial survival and transmission
**Pathogenic role**	
	**NTBF and ETBF strains**
	Show an expansive pangenome characterized by extensive genetic diversity
	Show the capability to evade the host immune response Show resistance/multi-resistance to antibiotics
	**NTBF strains**
	Contribute to the development and progression of metabolic disorders (obesity and diabetes) and atherosclerotic cardiovascular disease
	Induce intra-abdominal abscess development and intrahepatic cholestasis (ICP)
	**ETBF strains**
	Cause gastrointestinal infection and intestinal and extra-intestinal abscesses formation
	Promote chronic inflammation, neurodegeneration and carcinogenesis

## Data Availability

No new data were created or analyzed in this study. Data sharing is not applicable to this article.
